# Systems Pharmacological Approach to the Effect of* Bulsu-san* Promoting Parturition

**DOI:** 10.1155/2017/7236436

**Published:** 2017-10-29

**Authors:** Su Yeon Suh, Won G. An

**Affiliations:** Department of Pharmacology, School of Korean Medicine, Pusan National University, Yangsan, Gyeongnam 50612, Republic of Korea

## Abstract

*Bulsu-san* (BSS) has been commonly used in oriental medicine for pregnant women in East Asia. The purpose of this research was to elucidate the effect of BSS on ease of parturition using a systems-level in silico analytic approach. Research results show that BSS is highly connected to the parturition related pathways, biological processes, and organs. There were numerous interactions between most compounds of BSS and multiple target genes, and this was confirmed using herb-compound-target network, target-pathway network, and gene ontology analysis. Furthermore, the mRNA expression of relevant target genes of BSS was elevated significantly in related organ tissues, such as those of the uterus, placenta, fetus, hypothalamus, and pituitary gland. This study used a network analytical approach to demonstrate that Bulsu-san (BSS) is closely related to the parturition related pathways, biological processes, and organs. It is meaningful that this systems-level network analysis result strengthens the basis of clinical applications of BSS on ease of parturition.

## 1. Introduction

The name of* Bulsu-san* (BSS) originated from its therapeutic effects that help to promote easy labor as if being touched by merciful Buddha's hand [1]. BSS is composed of* Angelicae Sinensis* Radix (Danggui, DG) and* Cnidium officinale* Makino (Cheongung, CG), which is one of the most commonly used herb pairs in Traditional Medicine of East Asia and the usual component ratio is 2 : 3 (CG : DG) or 1 : 1 [2]. BSS is widely used in women's medicine in East Asia; its recognized therapeutic effects are as follows: removal of impure blood, blood making, easy parturition, acceleration of labor, elimination of dead fetus or placenta, amelioration of pain, nourishing blood, and promoting blood circulation [3].

What is more, recent experimental research on the CG-DG herb pair indicated that they affect the nourishment of blood [4], activate blood circulation, and prevent blood stasis [5]. In addition, the CG-DG herb pair showed significant inhibitory effects on the proliferation and protein synthesis of vascular smooth muscle cells [6]. It was suggested BSS could affect the activities of Akt kinase and eNOS by increasing intracellular Ca^2+^ and reducing ROS levels [7] and regulate menstruation and provide relief from pain by enabling the management of uterine smooth muscle contractions [8]. Although BSS has therapeutic effects on various pathological symptoms in pregnant or childbearing aged women, this research focused on the molecular mechanisms and impact of BSS on easing parturition and the acceleration of labor.

In terms of parturition onset, numerous studies have described the complex hormone interactions between estrogen, progesterone, oxytocin, corticosteroid, and prostaglandin. Among these, corticotrophin releasing hormone (CRH) is regarded as a trigger that initiates the labor [9]. The placenta releases substantial amounts of CRH, which stimulates the pituitary glands of both mother and fetus to secrete adrenocorticotropin hormone [10]. This in turn induces the release of estrogen precursor, which is converted into estrogen by the placenta that induces smooth muscle contraction [10]. Additionally, dilatation of cervical connective tissue and smooth muscle is induced by the following changes: a shift from progesterone to estrogen dominance, increased responsiveness to oxytocin via the upregulation of myometrial oxytocin receptor, increased prostaglandins synthesis in uterus, increased myometrial gap junction formation, decreased nitric oxide activity, and increased influx of calcium into myocyte [11].

**Figure 1:**
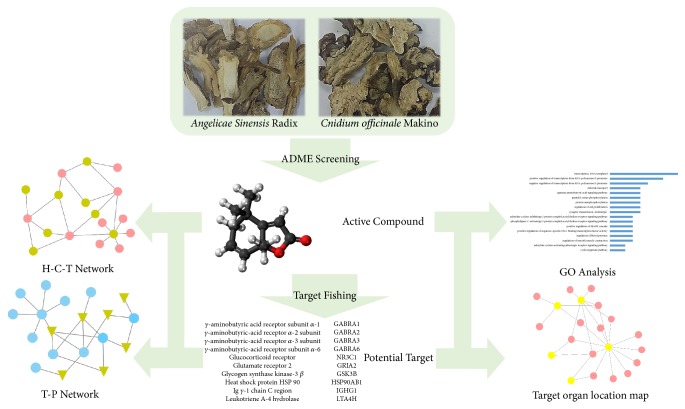
The workflow: the network pharmacological approach of Bulsu-san (BSS), namely, active compounds screening, target fishing, network analysis, and relevant organ location mapping was performed in this study.

The hypothesis of this study was that BSS may promote the positive-feedback of hormone loops as well as a series of myometrial and cervical changes to ease parturition and safely accelerate labor. A network based in silico approach was used to identify the effect of BSS on parturition related systems and the aim of this study was to elucidate the effect of BSS on the parturition by system-level analysis. The workflow of the network pharmacological study is summarized in Figure 1.

## 2. Material and Methods

### 2.1. Identification of Active Compounds

Compounds in CG and DG were identified using a phytochemical database that is the Traditional Chinese Medicine Systems Pharmacology (TCMSP, http://ibts.hkbu.edu.hk/LSP/tcmsp.php). We applied parameters related to absorption, distribution, metabolism, and excretion (ADME), namely, human drug-likeness (DL) [12], oral bioavailability (OB) [13], and Caco-2 permeability (Caco-2) to screen the Potential active compounds in BSS [14].

#### 2.1.1. Drug-Likeness Evaluation

DL helps filter “drug-like” compounds in oriental herbs, as DL represents a qualitative concept for valuations based on how “drug-like” a prospective compound is [15]. Accordingly, a high DL may lead to a greater possibility of therapeutic success, and compounds with a higher DL value are more likely to possess certain biological properties [16]. The calculations of DL in TCMSP database were based on Tanimoto coefficient formula [17] as follows:

(1) $$\begin{array}{c} F\left(\;A,B\;\right)=\frac{A\times B}{A^{2}+B^{2}-A\times B}, \end{array}$$

where *A* represents the molecular parameters of herbal compounds and *B* is the average molecular parameters of all compounds in the Drugbank database (http://www.drugbank.ca/) [18]. In the present study, we excluded compounds with a DL of <0.08. Other previous researches of herbal formulas set a higher threshold in the range of 0.1 to 0.18. However, we found out that most compounds of DG have low DL. In detail, only 36 compounds of 125 in DG show higher or equal DL value than 0.08. For this reason, this study sets a lower threshold of DL than other previous researches to see the most potential targets of BSS.

#### 2.1.2. Oral Bioavailability (OB) Prediction

OB is defined as the ratio of active compounds' absorption into the systemic circulation, which represents the convergence of the ADME process [13]. OB values are dependent on drug dissolution in the gastrointestinal (GI) tract and hepatic and intestinal first-pass metabolism, as well as on intestinal membrane permeation, which makes it a major pharmacokinetic parameter for drug evaluations [16]. In this study, the OB threshold was set as ≥15%.

#### 2.1.3. Caco-2 Permeability Screening

Caco-2 permeability is used to predict the absorption of an orally administered drug [14]. Surface absorptivity of the small intestine is maximized with the presence of villi and microvilli, for this reason most orally administered drug absorption occurs in the small intestine [19]. Moreover, the movement of orally administered drugs across the intestinal epithelial barrier determines the rate and extent of human absorption and ultimately affects drug bioavailability [20]. In the present study, compounds with OB, DL and Caco-2 values of greater than 15%, 0.08, and >−0.4, respectively, were regarded as active compounds and subjected to further analysis.

#### 2.1.4. Lipinski's Rule (LR) Screening

In addition, the screening standard used was defined based on Lipinski's rule (LR), which identifies druggable compounds as having molecular weight (MW) of ≤500 Da (MW ≤ 500), chemical composition with ≤5 hydrogen-bond donors, ≤10 hydrogen-bond acceptors, and an octanol-water partition coefficient, AlogP of ≤ 5 [21]. AlogP can be used to estimate local hydrophobicity, to produce molecular hydrophobicity maps, and to evaluate hydrophobic interactions in protein-ligand complexes [22]. Hdon and Hacc are the number of possible hydrogen-bond donors and acceptors, and the hydrogen-bonding capacity of a drug solute is recognized as a crucial determinant of permeability; moreover high hydrogen-bonding potential is often related to low permeability and absorption [23]. Eventually, in the present study, we selected active compounds satisfying the following criteria: OB ≥ 15%; DL ≥ 0.08; Caco-2 ≥ −0.4; MW ≤ 500; H-bond donors ≤ 5; H-bond acceptors ≤ 10; AlogP ≤ 5.

### 2.2. Target Fishing

Aside from filtering active compounds, we also sought to identify the molecular targets of these active compounds. Compound-target interaction profiles were established based on a systematic prediction of multiple drug-target interactions tool which employs random forest (RF) and support vector machine (SVM) methods and integrates chemical, genomic, and pharmacological information for drug targeting and discovery on a large scale [24]. Compound-target interactions satisfying SVM score ≥ 0.8 and RF score ≥ 0.7 were selected for further study. Additionally, filtered compound-target interaction profile mapping was performed using the UniProt database (http://www.uniprot.org/) [25].

### 2.3. Gene Ontology (GO) Analysis

Biological process (BP) of gene ontology (GO) analysis was employed to determine the biological properties of target genes [26]. GO annotation indicates the possibility of direct statistical analysis on gene function information. In this research, GO BP terms with *P* values < 0.01 were employed and the data was collected using the DAVID 6.8 Gene Functional Classification Tool (http://david.abcc.ncifcrf.gov/).

### 2.4. Network Construction and Analysis

In order to understand the multiscale interactions between the active compounds of BSS and targets, two types of networks were built: (1) the herb-compound-target network (H-C-T network), in which nodes represent either compounds, target genes, or herbs and edges indicate herb-compound-target connections; and (2) the target-pathway network (T-P network) to extract the pathways from KEGG database (http://www.genome.jp/kegg/), and the terms highly associated with parturition with *P* values < 0.05 were selected as the related pathways of targets in this work. Related targets were mapped onto relevant pathways, which resulted in the T-P network. Both networks were generated in Cytoscape 3.5.1, an open-source biological network visualization and data integration software package [27].

### 2.5. Target Organ Location Map

Tissue-specific patterns of mRNA expression can indicate important associations with biological events or gene functions [28]. To explore the beneficial effects of BSS during parturition, it is important that the tissue mRNA expression profiles of target genes at the organ level be known [29]. The target organ location map was built according to the Dataset: GeneAtlas U133A, gcrma (http://biogps.org). BioGPS database provides expression data acquired by direct measurements of gene expression obtained by microarrays analysis [30]. First, the mRNA expression patterns of each target gene in 176 parts of organ tissues were obtained. Second, average values were calculated for each gene. Third, frequency of above average mRNA expression tissue organs was inspected. Forth, based on the result from the third step and parturition mechanism theory, mRNA expression data of relevant organ tissues were extracted and categorized into 6 groups, namely, uterus and/or uterus corpus, fetus and/or placenta, hypothalamus and/or pituitary, smooth muscle, and whole blood.

## 3. Results

### 3.1. Identification of Active Compounds

314 compounds of BSS were identified, including 189 molecules in CG and 125 in DG (as shown in Supplementary Material Table S1 in Supplementary Material available online at https://doi.org/10.1155/2017/7236436) and active compounds met the criteria OB ≥ 15%, Caco-2 ≥ −0.4, and DL ≥ 0.08, as well as the standards of Lipinski's rule (LR) (as shown in Table 1). In detail, 60 active compounds were initially chosen, but 8 compounds were present in both herbs, namely, 3-butylidene-7-hydroxyphthalide, adenine, BdPh, beta-selinene, palmitic acid, senkyunolide-C, senkyunolide-D, and senkyunolide-E, and 14 had no target protein information and were thus excluded from the list of active compounds, whereas 27 compounds with lower ADME properties than above thresholds were included, which were reported to be related to oxytocin. In total, 65 active compounds were filtered.

Although ligustilide and ferulic acid have a DL of <0.08, both were included in this study. Since ligustilide (C12, DL = 0.07, OB = 53.72, Caco-2 = 1.3) was reported to be the main compound of DG in uterine contraction [31], and ferulic acid (C42, DL = 0.06, OB = 54.97, Caco-2 = 0.53) has been reported to be useful for the treatment of vascular diseases [6, 32] and blood deficiency syndrome [33] in China and to suppress inflammatory responses and tumor progression [34]. Some other compounds also have been shown experimentally to have various biological activities; for example, crysophanol (C42, DL = 0.21, OB = 18.64, Caco-2 = 0.62) can be used to treat menorrhagia and thrombocytopenia [35]. Perlolyrine (C52, DL = 0.27, OB = 65.95, Caco-2 = 0.88) was confirmed to have a protective effect on injured human umbilical vein endothelial cells [36], and myricanone (C48, DL = 0.51, OB = 57.61, Caco-2 = 0.67) was found to best inhibit mouse skin tumor progression [37].

### 3.2. Target Fishing

The 65 active compounds interact with 185 target proteins, as shown in Table 2; in other words, on average, each compound on average interacts with 2.85 target proteins. This result confirms the polypharmacological character of oriental medicine and demonstrates the synergistic effects of multiple compounds on multiple targets [38]. Different compounds in CG and DG can directly affect common targets, for example, the target protein “calmodulin (CALM1)” interacts with crysophanol from CG and coniferyl ferulate from DG at the same time, which implies the synergetic or cumulative effects of herbal medicine.

### 3.3. GO Analysis

**Figure 2:**
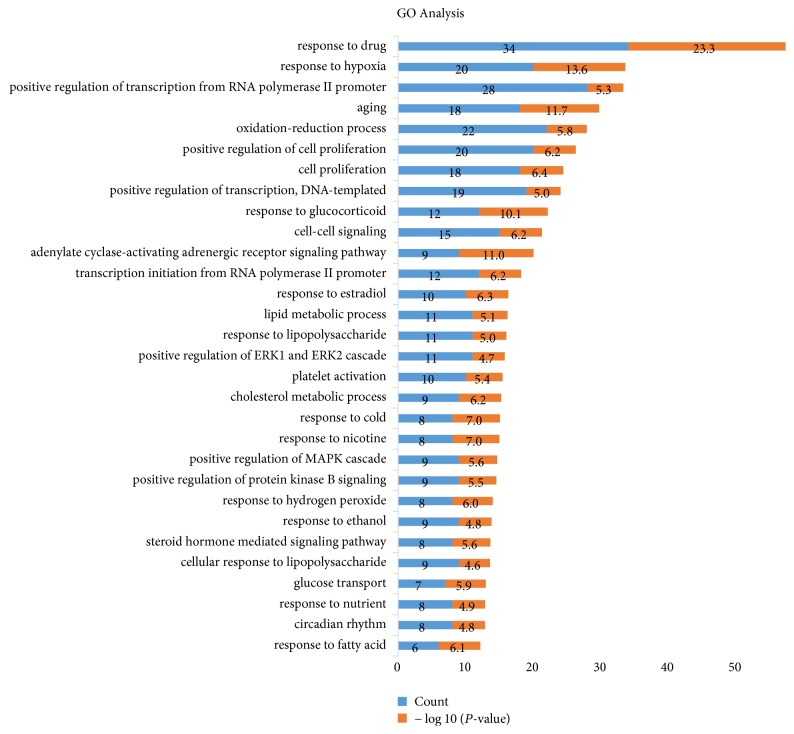
GO analysis: 30 enriched biological process (BP) of gene ontology (GO) terms sorted by *P* value < 0.01 and gene counts are displayed. The *y*-axis represents enriched biological process (BP) terms for the target genes, and the *x*-axis shows gene counts and −log⁡10 (*P* value).

397 biological process terms with *P* values of <0.01 were sorted using the functional annotation chart of the DAVID 6.8 Gene Functional Classification Tool, based on 185 filtered target genes, and *P* values were adjusted using the Benjamini-Hochberg method. 30 enriched GO BP terms extracted by *P* value and gene counts are displayed in Figure 2. It is meaningful that most of the target genes are significantly related to the various BP involved in parturition. For instance, 30 extracted GO BP terms include “MAPK signaling pathways,” “steroid hormone mediated signaling pathway,” “response to glucocorticoid,” “response to estradiol,” and “positive regulation of ERK1 and ERK2 cascade.” “MAPK signaling pathways” were reported to be activated in human uterine cervical ripening during parturition [39]. “Steroid hormone mediated signaling pathway” is highly related to parturition process as estrogen and progesterone play important roles in pregnancy and parturition, and estrogen induceS the principal stimulatory myometrial contractility [40]. Also, estradiol takes key place in parturition process [41]. It was identified that increased ERK activation is observed at the onset of labor, and it promotes myometrial contractility and development of parturition [42, 43]. To sum up, the target genes of BSS are highly associated with the biological process (BP) of parturition.

### 3.4. Network Construction and Analysis

**Figure 3:**
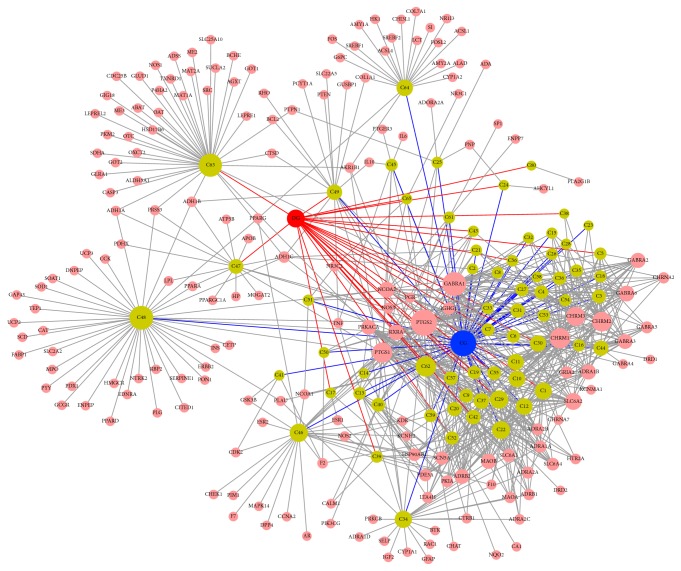
H-C-T network: herb-compound-target (H-C-T) network demonstrated multicompound, multitarget property of BSS. In this network, red and blue nodes represent herbs, green nodes show compounds, and pink nodes indicate targets and node size is relative to the degree and edges demonstrate interactions between nodes.

**Figure 4:**
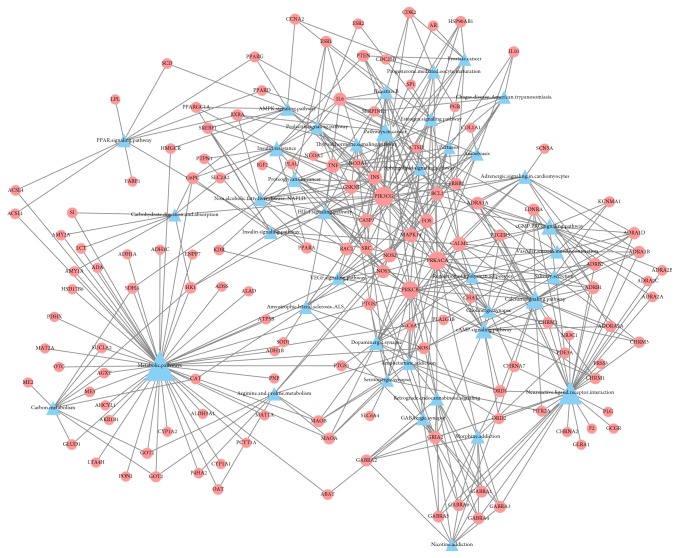
T-P network: in target-pathway (T-P) network, circular nodes represent compounds and triangles indicate pathways. Node size is relative to the degree and edges demonstrate interactions between nodes.

Network analysis is an efficient tool for visualizing and understanding multiple targeted drug actions and demonstrates drug actions within the context of the whole genome [44, 45]. For a better insight of therapeutic impacts, H-C-T and T-P networks were constructed and displayed in Figures 3 and 4, respectively. In the H-C-T network, nodes represent herb names, compounds, and targets. Also in the T-P network, circular nodes represent targets and triangle nodes represent pathways. Besides node size is relative to the degree and edges show interactions between nodes.

H-C-T network confirmed that there were 739 interactions between 185 targets and 65 active compounds of CG and DG: oleic acid (C48, degree = 42) with the highest number of interactions with targets, followed by succinic acid (C63, degree = 40) and stigmasterol (C62, degree = 37). It shows that single molecules target multiple receptors [46]. Also, some compounds from CG and DG were found to share common targets. Likewise, prostaglandin G/H synthase 2 (PTGS2, degree = 56) displayed the most affinitive connections with compounds, followed by gamma-aminobutyric acid receptor subunit alpha-1 (GABRA1, degree = 48), prostaglandin G/H synthase 1 (PTGS1, degree = 37), and muscarinic acetylcholine receptor M1 (CHRM1, degree = 37). Except for C60 (PLA2G1B, degree = 1), the rest of the 64 active compounds are connected with more than one target; likewise, 73 (39.5%) target genes out of 185 interacted with more than one compound. This result demonstrates the multicompounds and multitarget properties of herbal compounds and there was a report that compounds with multiple targets could have greater therapeutic efficacy [47].

In addition, the top 40 pathways were extracted based on gene counts and *P* value (<0.05), and *P* value was adjusted by Benjamini-Hochberg method. T-P network using relevant targets of herbal compounds is demonstrated in Figure 4. There were 485 interactions between the top 40 pathways and 135 of 185 target genes. “Metabolic pathways” (degree = 49) and “neuroactive ligand-receptor interaction pathway” (degree = 32) had the highest and the second highest numbers of connections with the targets, followed by “calcium signaling” (degree = 21), “cAMP signaling pathway” (degree = 17), and “cGMP PKG signaling pathway” (degree = 15). These are compelling results that parturition processes are the complex hormone interactions and it is well known that calcium signals within the myometrium are pivotal for uterine contractions [48]. In the same manner, some target genes demonstrated higher degree centrality with top 40 pathways, namely, PI3-kinase subunit gamma (PIK3CG, degree = 23), cAMP-dependent protein kinase catalytic subunit alpha (PRKACA, degree = 20), protein kinase C beta type (PRKCB, degree = 18), and calmodulin (CALM1, degree = 11). We can confirm the same result in the previous researches. For instance, PI3-kinase subunit gamma plays the key role in regulating cAMP, calcium cycling, and beta-adrenergic signaling [49]. Moreover, during the labor, calmodulin-calcium complex activates myosin light-chain kinase, which causes the generation of ATPase activity; eventually, uterine contraction is promoted [50].

H-C-T network explains the multitarget, multicompounds properties and accumulates effect of herbal medicines and T-P network shows that target genes of BSS are highly related to the pathway associated with parturition process.

### 3.5. Target Organ Location Map

**Figure 5:**
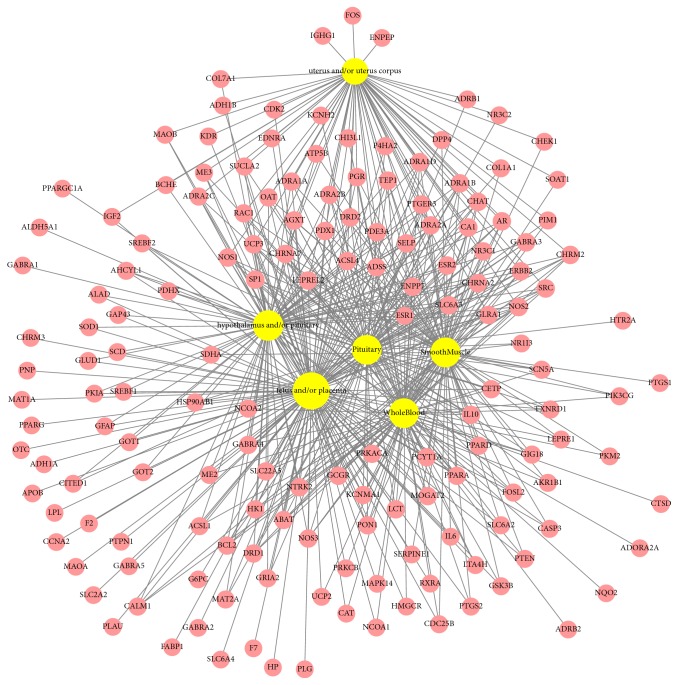
Target organ location map: it shows that tissue-specific patterns of mRNA expression are highly active in relative organs of parturition process such as uterus, fetus, placenta, hypothalamus, pituitary, and smooth muscle. Yellow nodes show compounds and pink nodes indicate targets and node size is relative to the degree and edges demonstrate interactions between nodes.

It is important to confirm the tissue mRNA expression profiles of the target genes at the organ level to identify the effects of BSS on parturition. Since there was no mRNA expression information in BioGPS of muscarinic acetylcholine receptor M1 (CHRM1), putative beta-glucuronidase-like protein SMA3 (GUSBP1), and retinol-binding protein 2 (RBP2), excluding these 3 targets from 185 filtered targets, totally 182 genes mRNA expression profiles were analyzed in this study. There were 519 interactions between target genes and organ locations. The networks of target genes tissue mRNA expression profiles and compounds of BSS are shown in Figure 5.

As a result, 159 of 182 target genes displayed beyond average mRNA expression in relevant organ tissues, such as uterus and/or uterus corpus, fetus and/or placenta, hypothalamus and/or pituitary, smooth muscle, and whole blood. The rest of 23 genes of 182 targets did not display above average mRNA expression in above organ tissues, for example, gamma-aminobutyric acid receptor subunit alpha-6 (GABRA6) and coagulation factor X (F10).

Nevertheless, most genes of 159 demonstrated high expression patterns in several organs of parturition related tissues at the same time. In detail, 60 genes showed most significant mRNA expression in the uterus and/or uterus corpus group, 130 for placenta and/or fetus, 86 for hypothalamus and/or pituitary, 82 for smooth muscle, 80 for pituitary, and 81 for whole blood. Besides, 30 of 159 genes showed expression in all of 6 groups. For instance, muscarinic acetylcholine receptor M2 (CHRM2), neuronal acetylcholine receptor subunit *α*-2 (CHRNA2), gamma-aminobutyric acid receptor subunit alpha-3 (GABRA3), NO synthase, inducible (NOS2), cGMP-inhibited 3′,5′-cyclic phosphodiesterase A (PDE3A), and sodium-dependent dopamine transporter (SLC6A3) recorded beyond average mRNA expression in all six groups. Furthermore, 79% of targets were expressed in two or more organ tissues, which suggests that those organs and target genes of BSS are closely correlated.

## 4. Discussion

In this study, network pharmacology method with DL, OB, Caco-2, and LR evaluation, multiple drug-target prediction, network analysis, and relevant organ location mapping was used to explain the targets of BSS in relation to the parturition process. There is no denying that network based analysis is powerful approach for identifying the actions of multitargeting herbal medicines at the systems level and our study shows target genes of BSS are strongly connected to parturition related pathways, biological processes, and organs. It was confirmed that 98% of the active compounds of BSS were interacted with more than two targets and 39.5% of the targets related to more than one compound. The synergetic multitarget properties of BSS were visualized, but further discussion about differentiated drug action based on degree centrality and simultaneous targeting effect of more than one compound is required [51]. Also, detailed potential pathways of BSS should be explored deeply in the future.

Similar findings were identified in a few RCT researches in China that using BSS in induction of labor can reduce the delivery time, the amount of bleeding, and the residual rate of placenta [52, 53]. In addition, BSS targets six genes of GABA receptor and NOS, which was reported to be related oxytocin neurons at the time of parturition in rats [54]. Also, BSS targets NOS and NO (nitric oxide) which are involved in the regulation of uterine contractility during pregnancy and is a key factor for the onset of labor [55], and iNOS (inducible nitric oxide synthase) can be upregulated accordantly by similar inflammatory mediators during ripening [11].

In fact, rather than DG,* Angelicae Gigantis Radix* (Danggwi, AGR) grows naturally in Korea; for that reason, the combination of AGR and CG is commonly used as BSS in Korea. Instead, DG is named as Chinese Danggwi for accurate classification in Korea. Several studies have shown AGR is differs from DG in terms of its main active constituents and genetic form. AGR is mainly composed of water soluble polysaccharide but coumarin, which is liposoluble including nodakenin (1), peucedanone (2), marmesin (3), decursinol (4), 7-hydroxy-6-(2R-hydroxy-3-methylbut-3-enyl) coumarin (5), demethylsuberosin (6), decursin (7), decursinol angelate (8), and isoimperatorin (9) [56]. Of these, decursin and its isomer decursinol angelate have been reported to be the active compounds in AGR [57]. It was identified in the experimental studies that AGR and DG act via different mechanisms in the cardiovascular, central nervous system, and anticancer activity but both have similar pharmacological effects [57]. Since the compositions of DG and AGR differ, further study on BSS with AGR is required. Currently, BSS is commonly prescribed to treat cerebra vascular and cardiovascular diseases in China [33], but, in Korea, BSS is widely applied in obstetrics.

The similarity between cervical ripening during parturition and inflammatory reaction has been pointed out in earlier studies; this has been attributed to the induction of leukocyte migration into tissue, thus promoting cervical remodeling and parturition by estrogen [58]. Further study is needed in terms of the effect of BSS on inflammatory reactions and parturition.

Furthermore, the CG-DG herb pair has other names, such as, Gunggui-tang (weight ratios of 2 : 3 or 1 : 1), Ogeum-san (1 : 1), Iphyo-san (1 : 1), and Sinmyo Bulsu-san (1 : 2), those are prepared at different weight ratios [3]. Accordingly, weight ratio should be determined based on considerations of targeted symptoms for relevant clinical applications.

## 5. Conclusion

This study results show that* Bulsu-san* (BSS) is highly connected to the parturition related pathways, biological processes, and organs. Most compounds in BSS work together with multiple target genes in a synergetic way, and this was confirmed using herb-compound-target network and target-pathway network analysis. The mRNA expression of relevant target genes of BSS was elevated significantly in parturition related organ tissues, such as those of the uterus, placenta, fetus, hypothalamus, and pituitary gland.

This study employed the network analytical methods to show the multicompound, multitarget properties of BSS. The results not only support clinical applications of BSS on easing childbirth but also suggest the related target genes and pathways of BSS on promoting parturition according to a systems-level in silico analytic approach. However, detailed mechanisms and other functions of BSS should be discussed further.

## Supplementary Material

Table S1: 314 Compounds of BSS (189 molecules of CG and 125 of DG).

## Figures and Tables

**Table 1:** 65 Potential active compounds of BSS (compound with *∗* was present in both herbs).

ID	Active compounds	OB (%)	Caco-2	DL	Herb
C1	()-alpha-Terpineol	46.3	1.28	0.03	DG
C2	()-Aromadendrene	55.74	1.81	0.1	CG
C3	()-Terpinen-4-ol	81.41	1.36	0.03	CG
C4	(+)-alpha-Funebrene	52.87	1.79	0.1	CG
C5	(+)-Ledol	16.96	1.43	0.12	DG
C6	(1R,5R,7S)-4,7-Dimethyl-7-(4-methylpent-3-enyl)bicyclo[3.1.1]hept-3-ene	16.23	1.86	0.09	CG
C7	(1S,4aR,8aR)-1-Isopropyl-7-methyl-4-methylene-2,3,4a,5,6,8a-hexahydro-1H-naphthalene	19.8	1.86	0.08	DG
C8	(1S,4E,8E,10R)-4,8,11,11-tetramethylbicyclo[8.1.0]undeca-4,8-diene	21.69	1.86	0.08	CG
C9	(3E)-3-butylidene-7-hydroxy-2-benzofuran-1-one	42.17	1.03	0.08	DG
C10	(L)-alpha-Terpineol	48.8	1.39	0.03	CG
C11	(R)-Linalool	39.8	1.33	0.02	CG
C12	(Z)-Ligustilide	53.72	1.3	0.07	CG
C13	1-Acetyl-beta-carboline	67.12	1.18	0.13	CG
C14	1-beta-Ethylacrylate-7-aldehyde-beta-carboline	28.53	0.45	0.31	CG
C15	1H-Cycloprop(e)azulen-7-ol, decahydro-1,1,7-trimethyl-4-methylene-, (1aR-(1aalpha,4aalpha,7beta,7abeta,7balpha))-	82.33	1.37	0.12	CG
C16	1-Terpineol	49.83	1.24	0.03	CG
C17	2,6-Di(phenyl)thiopyran-4-thione	69.13	1.74	0.15	DG
C18	2-[(2S,5S,6S)-6,10-Dimethylspiro[4.5]dec-9-en-2-yl]propan-2-ol	37.62	1.44	0.09	CG
C19^*∗*^	3-Butylidene-7-hydroxyphthalide	62.68	1	0.08	CG&DG
C20	4,7-Dihydroxy-3-butylphthalide	106.09	0.69	0.1	CG
C21	49070_FLUKA	85.51	1.29	0.12	CG
C22	4-Hydroxy-3-butylphthalide	70.31	0.9	0.08	CG
C23	58870_FLUKA	49.01	1.82	0.1	CG
C24^*∗*^	Adenine	62.81	−0.3	0.03	CG&DG
C25	ADO	15.98	−1.56	0.18	CG
C26	alpha-Cubebene	16.73	1.83	0.11	CG
C27	alpha-Selinene	31.81	1.82	0.1	CG
C28	Aromadendrene oxide 2	65.1	1.56	0.14	CG
C29^*∗*^	BdPh	42.44	1.32	0.07	CG&DG
C30	beta-Chamigrene	31.99	1.82	0.08	DG
C31^*∗*^	beta-Selinene	24.39	1.83	0.08	CG&DG
C32	beta-Cubebene	32.16	1.82	0.11	CG
C33	Cadinene	17.12	1.88	0.08	DG
C34	Caffeic acid	25.76	0.21	0.05	CG
C35	Carotol	149.03	1.46	0.09	CG
C36	Cedrene	51.14	1.82	0.11	CG
C37	Chuanxiongol	22.19	0.94	0.1	CG
C38	cis-Thujopsene	56.43	1.84	0.12	DG
C39	Coniferyl ferulate	4.54	0.71	0.39	DG
C40	Crysophanol	18.64	0.62	0.21	CG
C41	FA	68.96	−1.5	0.71	CG
C42	Ferulic acid (CIS)	54.97	0.53	0.06	DG
C43	InChI=1/C15H24/c1-10-7-8-15-9-12(10)14(3,4)13(15)6-5-11(15)2/h7,11-13H,5-6,8-9H2,1-4H	55.56	1.79	0.1	DG
C44	L-Bornyl acetate	65.52	1.29	0.08	CG
C45	Methyl palmitate	18.09	1.37	0.12	CG
C46	Myricanone	40.6	0.67	0.51	CG
C47	Nicotinic acid	47.65	0.34	0.02	DG
C48	Oleic acid	33.13	1.17	0.14	CG
C49^*∗*^	Palmitic acid	19.3	1.09	0.1	CG&DG
C50	Perlolyrine	65.95	0.88	0.27	CG
C51	PLO	14.07	0.69	0.43	CG
C52	Scopoletol	27.77	0.71	0.08	DG
C53	Senkyunolide A	26.56	1.3	0.07	CG
C54	Senkyunolide G	39.52	0.63	0.08	CG
C55^*∗*^	Senkyunolide-C	46.8	0.87	0.08	CG&DG
C56^*∗*^	Senkyunolide-D	79.13	0.12	0.1	CG&DG
C57^*∗*^	Senkyunolide-E	34.4	0.55	0.08	CG&DG
C58	Senkyunolide-K	61.75	0.52	0.08	CG
C59	Sinapic acid	64.15	0.48	0.08	CG
C60	Sphingomyelin	0.31	−0.46	0.51	DG
C61	Stearic acid	17.83	1.15	0.14	CG
C62	Stigmasterol	43.83	1.44	0.76	DG
C63	Succinic acid	29.62	−0.44	0.01	DG
C64	Sucrose	7.17	−2.89	0.23	CG
C65	Wallichilide	42.31	0.82	0.71	CG

**Table 2:** Related targets of potential compounds in BSS.

UniProt ID	Target name	Gene Name
P80404	4-aminobutyrate aminotransferase, mitochondrial	ABAT
P33121	Long-chain-fatty-acid-* *-CoA ligase 1	ACSL1
O60488	Long-chain-fatty-acid-* *-CoA ligase 4	ACSL4
P00813	Adenosine deaminase	ADA
P07327	Alcohol dehydrogenase 1A	ADH1A
P00325	Alcohol dehydrogenase 1B	ADH1B
P00326	Alcohol dehydrogenase 1C	ADH1C
P29274	Adenosine A2a receptor	ADORA2A
P35348	Alpha-1A adrenergic receptor	ADRA1A
P35368	Alpha-1B adrenergic receptor	ADRA1B
P25100	Alpha-1D adrenergic receptor	ADRA1D
P08913	Alpha-2A adrenergic receptor	ADRA2A
P18089	Alpha-2B adrenergic receptor	ADRA2B
P18825	Alpha-2C adrenergic receptor	ADRA2C
P08588	Beta-1 adrenergic receptor	ADRB1
P07550	Beta-2 adrenergic receptor	ADRB2
Q5SY84	Adenylosuccinate synthetase	ADSS
P21549	Serine-* *-pyruvate aminotransferase	AGXT
O43865	Putative adenosylhomocysteinase 2	AHCYL1
P15121	Aldose reductase	AKR1B1
P13716	Delta-aminolevulinic acid dehydratase	ALAD
P51649	Succinate semialdehyde dehydrogenase, mitochondrial	ALDH5A1
P04745	Alpha-amylase 1	AMY1A
P04746	Pancreatic alpha-amylase	AMY2A
P04114	Apolipoprotein B-100	APOB
P10275	Androgen receptor	AR
P06576	ATP synthase subunit beta, mitochondrial	ATP5B
P06276	Cholinesterase	BCHE
P10415	Apoptosis regulator Bcl-2	BCL2
Q06187	Tyrosine-protein kinase BTK	BTK
P00915	Carbonic anhydrase I	CA1
P62158	Calmodulin	CALM1
P42574	Caspase-3	CASP3
P04040	Catalase	CAT
P06307	Cholecystokinin	CCK
P20248	Cyclin-A2	CCNA2
P30305	M-phase inducer phosphatase 2	CDC25B
P24941	Cell division protein kinase 2	CDK2
P11597	Cholesteryl ester transfer protein	CETP
P28329	Choline O-acetyltransferase	CHAT
O14757	Serine/threonine-protein kinase Chk1	CHEK1
P36222	Chitinase-3-like protein 1	CHI3L1
P11229	Muscarinic acetylcholine receptor M1	CHRM1
P08172	Muscarinic acetylcholine receptor M2	CHRM2
P20309	Muscarinic acetylcholine receptor M3	CHRM3
Q15822	Neuronal acetylcholine receptor subunit alpha-2	CHRNA2
P36544	Neuronal acetylcholine receptor protein, alpha-7 chain	CHRNA7
Q99966	Cbp/p300-interacting transactivator 1	CITED1
P02452	Collagen alpha-1(I) chain	COL1A1
Q02388	Collagen alpha-1(VII) chain	COL7A1
P17538	Chymotrypsinogen B	CTRB1
P07339	Cathepsin D	CTSD
P04798	Cytochrome P450 1A1	CYP1A1
P05177	Cytochrome P450 1A2	CYP1A2
Q9ULA0	Aspartyl aminopeptidase	DNPEP
P27487	Dipeptidyl peptidase IV	DPP4
P21728	Dopamine D1 receptor	DRD1
P14416	D(2) dopamine receptor	DRD2
P25101	Endothelin-1	EDNRA
Q07075	Glutamyl aminopeptidase	ENPEP
Q6UWV6	Ectonucleotide pyrophosphatase/phosphodiesterase family member 7	ENPP7
P04626	Receptor tyrosine-protein kinase erbB-2	ERBB2
P03372	Estrogen receptor	ESR1
Q92731	Estrogen receptor beta	ESR2
P00742	Coagulation factor Xa	F10
P00734	Thrombin	F2
P08709	Coagulation factor VII	F7
P07148	Fatty acid-binding protein, liver	FABP1
P01100	Proto-oncogene c-Fos	FOS
P15408	Fos-related antigen 2	FOSL2
P35575	Glucose-6-phosphatase	G6PC
P14867	Gamma-aminobutyric acid receptor subunit alpha-1	GABRA1
P47869	Gamma-aminobutyric-acid receptor alpha-2 subunit	GABRA2
P34903	Gamma-aminobutyric-acid receptor alpha-3 subunit	GABRA3
P48169	Gamma-aminobutyric-acid receptor subunit alpha-4	GABRA4
P31644	Gamma-aminobutyric-acid receptor alpha-5 subunit	GABRA5
Q16445	Gamma-aminobutyric-acid receptor subunit alpha-6	GABRA6
P17677	Neuromodulin	GAP43
P47871	Glucagon	GCGR
P14136	Glial fibrillary acidic protein	GFAP
Q2TU84	Growth-inhibiting protein 18	GIG18
P23415	Glycine receptor alpha-1 chain	GLRA1
P00367	Glutamate dehydrogenase 1, mitochondrial	GLUD1
P17174	Aspartate aminotransferase, cytoplasmic	GOT1
P00505	Aspartate aminotransferase, mitochondrial	GOT2
P42262	Glutamate receptor 2	GRIA2
P49841	Glycogen synthase kinase-3 beta	GSK3B
Q15486	Putative beta-glucuronidase-like protein SMA3	GUSBP1
P19367	Hexokinase-1	HK1
P04035	3-hydroxy-3-methylglutaryl-coenzyme A reductase	HMGCR
P00738	Haptoglobin	HP
O14756	Oxidoreductase	HSD17B6
P08238	Heat shock protein HSP 90	HSP90AB1
P28223	5-hydroxytryptamine 2A receptor	HTR2A
P01344	Insulin-like growth factor II	IGF2
P01857	Ig gamma-1 chain C region	IGHG1
P22301	Interleukin-10	IL10
P05231	Interleukin-6	IL6
P01308	Insulin	INS
Q12809	Potassium voltage-gated channel subfamily H member 2	KCNH2
Q12791	Calcium-activated potassium channel subunit alpha 1	KCNMA1
P35968	Vascular endothelial growth factor receptor 2	KDR
P09848	Lactase-phlorizin hydrolase	LCT
Q32P28	Prolyl 3-hydroxylase 1	LEPRE1
Q8IVL6	Prolyl 3-hydroxylase 3	LEPREL2
P06858	Lipoprotein lipase	LPL
P09960	Leukotriene A-4 hydrolase	LTA4H
P21397	Amine oxidase [flavin-containing] A	MAOA
P27338	Amine oxidase [flavin-containing] B	MAOB
Q16539	Mitogen-activated protein kinase 14	MAPK14
Q00266	S-adenosylmethionine synthetase isoform type-1	MAT1A
P31153	S-adenosylmethionine synthetase isoform type-2	MAT2A
P23368	NAD-dependent malic enzyme, mitochondrial	ME2
Q16798	NADP-dependent malic enzyme, mitochondrial	ME3
Q3SYC2	2-acylglycerol O-acyltransferase 2	MOGAT2
P05164	Myeloperoxidase	MPO
Q15788	Nuclear receptor coactivator 1	NCOA1
Q15596	Nuclear receptor coactivator 2	NCOA2
P29475	Nitric-oxide synthase, brain	NOS1
P35228	Nitric oxide synthase, inducible	NOS2
P29474	Nitric oxide synthase, endothelial	NOS3
P16083	NRH dehydrogenase [quinone] 2	NQO2
Q14994	Nuclear receptor subfamily 1 group I member 3	NR1I3
P04150	Glucocorticoid receptor	NR3C1
P08235	Mineralocorticoid receptor	NR3C2
Q16620	BDNF/NT-3 growth factors receptor	NTRK2
P04181	Ornithine aminotransferase, mitochondrial	OAT
P00480	Ornithine carbamoyltransferase, mitochondrial	OTC
Q9BYC2	Succinyl-CoA:3-ketoacid-coenzyme A transferase 2, mitochondrial	OXCT2
O15460	Prolyl 4-hydroxylase subunit alpha-2	P4HA2
P49585	Choline-phosphate cytidylyltransferase A	PCYT1A
Q14432	CGMP-inhibited 3′,5′-cyclic phosphodiesterase A	PDE3A
O00330	Pyruvate dehydrogenase protein X component, mitochondrial	PDHX
P52945	Pancreas/duodenum homeobox protein 1	PDX1
P06401	Progesterone receptor	PGR
P48736	Phosphatidylinositol-4,5-bisphosphate 3-kinase catalytic subunit, gamma isoform	PIK3CG
P11309	Proto-oncogene serine/threonine-protein kinase Pim-1	PIM1
P61925	cAMP-dependent protein kinase inhibitor alpha	PKIA
P14618	Pyruvate kinase isozymes M1/M2	PKM2
P04054	Phospholipase A2	PLA2G1B
P00749	Urokinase-type plasminogen activator	PLAU
P00747	Plasminogen	PLG
P00491	Purine nucleoside phosphorylase	PNP
P27169	Serum paraoxonase/arylesterase 1	PON1
Q07869	Peroxisome proliferator-activated receptor alpha	PPARA
Q03181	Peroxisome proliferator-activated receptor delta	PPARD
P37231	Peroxisome proliferator activated receptor gamma	PPARG
Q9UBK2	Peroxisome proliferator-activated receptor gamma coactivator 1-alpha	PPARGC1A
P17612	mRNA of PKA Catalytic Subunit C-alpha	PRKACA
P05771	Protein kinase C beta type	PRKCB
P35030	Trypsin-3	PRSS3
P60484	Phosphatidylinositol-3,4,5-trisphosphate 3-phosphatase and dual-specificity protein phosphatase PTEN	PTEN
P43115	Prostaglandin E2 receptor EP3 subtype	PTGER3
P23219	Prostaglandin G/H synthase 1	PTGS1
P35354	Prostaglandin G/H synthase 2	PTGS2
P18031	mRNA of Protein-tyrosine phosphatase, non-receptor type 1	PTPN1
P10082	Peptide YY	PYY
P63000	Ras-related C3 botulinum toxin substrate 1	RAC1
P50120	Retinol-binding protein 2	RBP2
P08100	Rhodopsin	RHO
P19793	Retinoic acid receptor RXR-alpha	RXRA
O00767	Acyl-CoA desaturase	SCD
Q14524	Sodium channel protein type 5 subunit alpha	SCN5A
P31040	Succinate dehydrogenase [ubiquinone] flavoprotein subunit, mitochondrial	SDHA
P16109	P-selectin	SELP
P05121	Plasminogen activator inhibitor 1	SERPINE1
P14410	Sucrase-isomaltase, intestinal	SI
O76082	Solute carrier family 22 member 5	SLC22A5
Q9UBX3	Mitochondrial dicarboxylate carrier	SLC25A10
P11168	Solute carrier family 2, facilitated glucose transporter member 2	SLC2A2
P23975	Sodium-dependent noradrenaline transporter	SLC6A2
Q01959	Sodium-dependent dopamine transporter	SLC6A3
P31645	Sodium-dependent serotonin transporter	SLC6A4
P35610	Sterol O-acyltransferase 1	SOAT1
P00441	Superoxide dismutase [Cu-Zn]	SOD1
P08047	Transcription factor Sp1	SP1
P12931	Proto-oncogene tyrosine-protein kinase SRC	SRC
P36956	Sterol regulatory element-binding protein 1	SREBF1
Q12772	Sterol regulatory element-binding protein 2	SREBF2
Q9P2R7	Succinyl-CoA ligase [ADP-forming] beta-chain, mitochondrial	SUCLA2
Q99973	Telomerase protein component 1	TEP1
P01375	Tumor necrosis factor	TNF
Q16881	Thioredoxin reductase, cytoplasmic	TXNRD1
P55851	Mitochondrial uncoupling protein 2	UCP2
P55916	Mitochondrial uncoupling protein 3	UCP3
